# LC-MS/MS Profiling of Blood Serum Reveals Disease-Enriched Peptides in Metabolic Syndrome

**DOI:** 10.3390/jpm16080425

**Published:** 2026-08-11

**Authors:** Alma Nurtazina, Valeriia Koss, Ivan Voitsekhovskiy, Maxat Toishimanov, Daulet Dautov, Kairat Karibayev, Victoria Shender, Georgij Arapidi

**Affiliations:** 1 Department of Epidemiology and Biostatistics, Semey Medical University, 071400 Semey, Kazakhstan; 2 Outpatient Clinic #1, Department of Internal Medicine and Cardiology, Semey Medical University, 071400 Semey, Kazakhstan; 3 Lopukhin Federal Research and Clinical Center of Physical-Chemical Medicine of Federal Medical Biological Agency, 119435 Moscow, Russia; victoria.shender@gmail.com (V.S.); arapidi@gmail.com (G.A.); 4 Center for Molecular and Cellular Biology, 121205 Moscow, Russia; 5 Faculty of Biology and Biotechnology, Al-Farabi Kazakh National University, 050040 Almaty, Kazakhstan; ivan.voitsekhovskiy@smu.edu.kz; 6 Kazakhstan-Japan Innovative Center, Kazakh National Agrarian Research University, 050010 Almaty, Kazakhstan; 7 Department of Propaedeutics of Internal Diseases, Asfendiyarov Kazakh National Medical University, 050012 Almaty, Kazakhstan; 8 Central Clinical Hospital, 050091 Almaty, Kazakhstan; 9 Shemyakin-Ovchinnikov Institute of Bioorganic Chemistry of the Russian Academy of Sciences, 117437 Moscow, Russia; 10 Moscow Center for Advanced Studies, 123592 Moscow, Russia

**Keywords:** metabolic syndrome, prediabetes, diabetes mellitus, LC-MS/MS, blood serum peptidomics, peptide biomarkers

## Abstract

**Background:** Metabolic syndrome (MetS) is a heterogeneous cardiometabolic group of conditions in which early disturbances of glucose homeostasis are not always adequately captured by routine clinical tests. **Methods:** In this pilot study, liquid chromatography–tandem mass spectrometry (LC-MS/MS)-based blood serum peptidomics was applied to identify circulating peptide signatures associated with MetS and progressive glycemic impairment. Serum samples from healthy donors and patients with MetS, including subgroups with normoglycemia, prediabetes, and diabetes mellitus, were subjected to peptide enrichment by ultrafiltration and solid-phase extraction prior to LC-MS/MS analysis. **Results:** Overall, 6754 unique peptides derived from 1820 precursor proteins were identified, and 32 peptides were significantly overrepresented in patients with MetS relative to healthy donors. Eight precursor proteins and five identified peptides showed significant positive correlations with glycemic stage, likely indicating progressive remodeling of the circulating serum peptidome during disease progression. A separate analysis of patients with prediabetes revealed additional candidate markers associated with early metabolic alterations. Moreover, multivariate logistic regression identified a four-peptide panel with good discriminatory performance for differentiating healthy donors from patients with MetS, yielding a leave-one-out cross-validated area under the curve of 0.91. **Conclusions:** Collectively, these findings indicate that serum peptidome profiling can reveal biologically and clinically relevant possible candidate biomarkers associated with MetS and early dysglycemia.

## 1. Introduction

Metabolic syndrome (MetS) is a multifactorial group of conditions defined by a constellation of interrelated cardiometabolic abnormalities, including abdominal obesity dyslipidemia and impaired glucose metabolism [1,2,3]. Owing to its high prevalence (MetS is estimated to affect approximately 20–25% of the adult population worldwide [4]) and its association with diabetes [5], cardiovascular complications [3], and increased mortality [6], MetS remains an important target for biomarker discovery.

Despite the widespread use of established clinical criteria, the diagnosis of MetS, particularly at early dysglycemic stages, remains challenging. Carbohydrate metabolism is commonly assessed by fasting plasma glucose, the oral glucose tolerance test (OGTT), and glycated hemoglobin (HbA1c), which reflects average blood glucose levels over the preceding 2–3 months. However, these measures are not fully interchangeable and often identify overlapping but not identical patient groups [7]. This limitation is especially evident in prediabetes, where concordance between HbA1c, fasting plasma glucose [8] and OGTT [9] is limited. Consequently, the use of an incomplete diagnostic panel may limit the detection of early disturbances in glucose metabolism [10]. The situation is further complicated by the fact that diagnostic thresholds and definitions of prediabetes are not uniformly standardized, allowing early metabolic alterations to remain undetected by routine screening approaches [11].

Blood is a particularly attractive source for the discovery of novel MetS biomarkers because it integrates molecular signals derived from multiple organs and tissues involved in disease pathogenesis, including adipose tissue, the liver, the vasculature, the immune system, and platelets [12]. Both plasma and serum may reflect systemic inflammation, vascular remodeling, and alterations in lipid and glucose metabolism [13]. In addition, blood sampling is minimally invasive and readily adaptable to clinical practice, making it especially suitable for translational biomarker research. Unlike the isolated assessment of individual clinical parameters, profiling a broad range of circulating proteins and endogenous peptides may enable detection of subtle and early pathological changes before the full clinical manifestation of disease becomes apparent [14].

LC-MS/MS-based approaches, including proteomics and peptidomics, offer considerable opportunities for identifying circulating molecular markers in plasma and serum [15]. These methods enable the simultaneous detection of large numbers of proteins and endogenous peptides, quantitative comparison across groups, and the identification of molecular signatures associated with disease presence or stage [16]. Similar strategies have already been successfully applied to the discovery of diagnostic and prognostic biomarkers in oncology [17], neurodegenerative disorders [18], cardiovascular diseases [19] and microbiota-related research [20], highlighting their potential relevance for MetS diagnosis as well. In this context, the identification of peptide- and protein-level markers in plasma and serum represents a well-justified approach for capturing both the general molecular features of MetS and earlier alterations associated with prediabetes and the progression of dysglycemia.

Compared with conventional proteomic profiling of intact proteins, endogenous peptidomics provides an additional and more dynamic layer of physiological information by reflecting proteolytic processing, protein turnover, and disease-associated changes in protease activity [21]. Owing to their small size, endogenous peptides may more readily pass from tissues and interstitial fluids into the circulation, potentially allowing tissue-associated metabolic alterations to become detectable before substantial changes in the circulating abundance of the corresponding intact proteins occur [21,22]. Furthermore, circulating peptides may associate with abundant carrier proteins, particularly albumin, which can protect them from rapid degradation and facilitate their persistence and detection in blood plasma or serum [23,24].

Despite extensive efforts to discover circulating protein biomarkers of metabolic and cardiovascular disorders, the translation of many proteomic candidates into clinically applicable markers has remained limited [25,26]. Previous studies of MetS have primarily focused on conventional clinical parameters and changes in intact plasma proteins, whereas the endogenous circulating peptide degradome and its relationship with progressive glycemic impairment remain comparatively underexplored.

In the present study, using a cohort of 23 donors in an LC-MS/MS-based peptidomics approach, we explored potential circulating peptide markers of MetS. We identified biologically plausible peptide candidates that not only distinguish patients with MetS from healthy donors but also discriminate individuals with prediabetes from healthy donors. Thus, this pilot study addresses the existing knowledge gap by characterizing naturally occurring serum peptide fragments associated with MetS and evaluating whether degradome-level signals may already be detectable at the early stage of dysglycemia.

## 2. Materials and Methods

### 2.1. Design of the Study

It was a pilot small-size case–control study.

### 2.2. Participants

In total, 23 participants were enrolled into the study. The data collection period was: 1 September 2024–30 December 2024. Eligible candidates were recruited via random sampling from outpatient clinics and included individuals of Kazakh ethnicity, aged 40 to 65 years, who met the diagnostic criteria for metabolic syndrome (MetS). Individuals were excluded from the study if they presented with coronary artery disease (CAD), systemic diseases, oncological malignancies, psychiatric disorders, or thyroid dysfunction. Additional exclusion criteria applied to concurrent statin, glucocorticoid therapy as well as pregnancy or lactation. Laboratory tests were collected during the same period.

Fasting serum samples from volunteers without the diagnosed disease and from patients with MetS were obtained at Semey Medical University (Semey, Kazakhstan). Putatively healthy donors followed a non-restricted mixed diet and were classified as healthy based on medical history and prospective clinical evaluation. Before blood sampling, each donor underwent physical and laboratory examination by a physician including assessment of vital signs and screening for acute infectious foci, with particular attention to the oropharynx. If any acute or chronic condition was detected either immediately before venipuncture or within one month after sampling, biospecimens from the respective donor were excluded from further analysis.

### 2.3. Ethical Considerations

The study protocol was reviewed and approved by the local Ethics Committee of Semey Medical University (approval no. G-041.06.01.06-2023, approval date 2 November 2023). Prior to enrollment, all participants received comprehensive information regarding the study’s objectives and significance. Written informed consent was obtained, ensuring that participation was entirely voluntary.

### 2.4. Diagnostic Criteria and Classifications

Patients with MetS were classified as having MetS according to the harmonized criteria of the International Diabetes Federation (IDF) and the American Heart Association/National Heart, Lung, and Blood Institute (AHA/NHLBI, 2009). MetS was defined by the presence of at least three of the following five components: (1) waist circumference (WC) ≥94 cm in men or ≥80 cm in women; (2) serum triglycerides (TG) ≥1.7 mmol/L; (3) high-density lipoprotein cholesterol (HDL-C) ≤ 1.0 mmol/L in men or ≤1.3 mmol/L in women; (4) systolic blood pressure (SBP) > 130 mmHg and/or diastolic blood pressure (DBP ≥ 85 mmHg, or ongoing antihypertensive therapy; and (5) fasting plasma glucose >100 mg/dL (5.6 mmol/L) or a prior diagnosis of type 2 diabetes mellitus (T2DM).

In this study patients with T2DM were newly diagnosed based on clinical symptoms, signs and laboratory parameters during the data collection period and had not received prior antidiabetic pharmacotherapy.

Fasting plasma glucose values were additionally used to stratify MetS patients into three glycemic categories: normoglycemia (3.5–5.5 mmol/L), prediabetes (5.6–6.9 mmol/L), and diabetes mellitus (≥7.0 mmol/L). In parallel, HbA1c was measured as a complementary indicator of glycemic status, with values < 5.7% considered normal, 5.7–6.4% classified as prediabetes, and ≥6.5% indicative of T2DM.

### 2.5. Blood Serum Collection

Venous blood was drawn from the cubital vein into Vacuette serum collection tubes (REF 456092; Greiner Bio-One, Kremsmünster, Austria). After allowing coagulation for up to 1 h at room temperature, samples were centrifuged for 15 min at 700 *g* at room temperature to obtain serum. The supernatant was separated, dispensed into 0.5 mL aliquots, and stored at −80 °C until further analysis.

### 2.6. Biochemistry

Serum levels of total cholesterol (TC), low-density lipoprotein cholesterol (LDL-C), high-density lipoprotein cholesterol (HDL-C), triglycerides (TG), fasting glucose, HbA1c, insulin, and creatinine were assayed in all participants using standardized protocols within a centralized laboratory.

### 2.7. Peptide Extraction from Blood Serum

Frozen serum aliquots were thawed at 4 °C, and 1.5 mL of 8 M guanidine hydrochloride (GuHCl; Sigma-Aldrich, St. Louis, Missouri, USA) was added to each sample. The mixture was incubated for 30 min at room temperature under continuous mixing to denature proteins and release peptides. Ultrafiltration was carried out using Vivaspin-2 centrifugal devices with a 10 kDa molecular, weight cutoff (Sartorius, Göttingen, Germany) on a 5804 R refrigerated centrifuge (Eppendorf, Hamburg, Germany).

Prior to sample loading, filters were conditioned by sequential centrifugation of 2 mL Milli-Q water and 2 mL 6 M GuHCl for 10 min at 8000 *g* at 4 °C. Subsequently, 2 mL of diluted serum was applied to each filter and centrifuged for 1–2 h at 8000 *g* at 4 °C until the retentate volume above the membrane was reduced to <200 µL. The concentrate was then resuspended in 700 µL of 6 M GuHCl and subjected to a second centrifugation step under identical conditions to further enrich peptides.

Desalting of the peptide-containing fraction was performed by reversed-phase solid-phase extraction using Discovery DSC-18 SPE Tube Supelco cartridges (Sigma-Aldrich, USA). To 2.5 mL of peptide solution, an equal volume of 2% methanol (MeOH; Merck, Darmstadt, Germany) in 0.2% trifluoroacetic acid (TFA; Sigma-Aldrich, USA) was added. The SPE columns were pre-conditioned with 1 mL MeOH and equilibrated with 2 mL of 1% MeOH in 0.1% TFA. Samples were loaded, washed with 2 mL of 1% MeOH in 0.1% TFA, and peptides were eluted with 400 µL of 50% MeOH in 0.1% TFA. The eluates were dried in a Concentrator plus centrifugal evaporator (Eppendorf, Germany) and stored at −80 °C. Immediately prior to LC-MS/MS analysis, dried residues were reconstituted in 15 µL of 1% MeOH in 0.1% TFA.

### 2.8. LC-MS/MS Analysis

Peptide analyses were performed on a Q Exactive HF-X mass spectrometer (Thermo Fisher Scientific, Waltham, USA) equipped with a NanoSpray Flex ion source and interfaced with an EASY-nLC 1200 nano-HPLC system configured in trap-elute mode. Solvent A consisted of water with 0.1% formic acid (FA, *v*/*v*), and solvent B of 80% acetonitrile (AcN) with 0.1% FA (*v*/*v*). Samples were first loaded onto a custom-packed trap column (100 μm × 10 mm, C18, 1.8 μm, 100 Å, Aeris Peptide, London, UK) at 2.5 μL/min for 10 min and then separated on a custom-packed analytical column (75 μm × 150 mm, C18, 1.8 μm, 100 Å, Aeris Peptide) at a flow rate of 300 nL/min. The gradient of solvent B was programmed as follows: 0–6% over 5 min, then 6–50% over 60 min.

Mass spectra were acquired in positive ion mode using a spray voltage of 2.2 kV, funnel RF level of 40, and a capillary temperature of 250 °C. Data-dependent acquisition (DDA) was employed with a full MS/dd-MS^2^ method including up to 20 dependent MS/MS scans per cycle. Full MS scans were acquired at a resolution of 120,000 (at *m*/*z* 200) with an AGC target of 3 × 10^6^ and a maximum injection time (IT) of 32 ms over an m/z range of 400–1600. For MS/MS scans, the AGC target was set to 2 × 10^5^ with an intensity threshold of 2.5 × 10^5^, and precursor isolation width was 1.4 m/z. Normalized collision energy was set to 27%. Precursor charge states from 2+ to 7+ were selected; peptide match was enabled and set to “preferred”, isotope exclusion was active, and dynamic exclusion was set to 45 s. All spectra were recorded in profile mode.

### 2.9. Peptide Identification

Raw data files acquired on the Q Exactive HF-X (Thermo Fisher Scientific, Waltham, USA) were converted to Mascot generic format (mgf) peak lists using MSConvert (ProteoWizard Software Foundation, release: 3.0.20287) with the following parameters: “--mgf --filter peakPicking true [1,2]”. The resulting peak lists were searched against the UniProt Knowledgebase (taxon human; downloaded 17 March 2026 with 227,375 protein sequences) concatenated with a reversed decoy set using MASCOT (version 2.5.1, Matrix Science Ltd., London, UK) and X! Tandem (ALANINE, 2017.02.01, The Global Proteome Machine Organization). For both search engines, precursor and fragment mass tolerances were set to 20 ppm and 50 ppm, respectively. No enzyme specificity and no fixed or variable post-translational modifications were specified in the search parameters.

Output files from the database searches were imported into Scaffold 5 (version 5.0.2, Proteome Software Inc., Portland, OR, USA) for peptide validation and downstream data handling, using standard probabilistic filtering to control identification confidence (5% FDR at the peptide level).

The mass spectrometry peptidomics data have been deposited to the ProteomeXchange Consortium via the PRIDE [27] partner repository with the dataset identifier PXD076281 and 10.6019/PXD076281.

### 2.10. Statistical Analysis

Peptide-spectrum matches (PSMs; spectral counts) were used as a semi-quantitative measure of the identified peptide abundance. For precursor protein analysis, PSMs of all peptides assigned to a given protein were summed. Group comparisons were performed using log2 fold change (log2FC) and *p*-values from the Mann–Whitney U test followed by Benjamini–Hochberg correction (*p*.adj).

Protein selection was performed in two stages. First, only precursor proteins whose peptides were identified in at least 10 patients with MetS were retained as candidates for comparison. Second, proteins were considered differentially abundant if they met thresholds of log2FC ≥ 1 and *p*.adj ≤ 0.05.

Peptide-level analysis was restricted to peptides derived from precursor proteins retained in the previous step. Candidate peptides were required to be identified in at least eight patients with MetS and to satisfy thresholds of log2FC ≥ 1 and *p*.adj ≤ 0.05.

To assess stage-associated patterns, Spearman correlation analysis was performed using an ordinal scale of 0, 1, 2, and 3 for healthy donors and for patients with normoglycemia, prediabetes, and diabetes mellitus, respectively. Correlation coefficients (rho) and Benjamini–Hochberg-adjusted *p*-values (*p*.adj) were calculated for both precursor protein signals and peptide abundances.

To construct a multiparametric classifier discriminating healthy donors from patients with MetS, multivariate logistic regression with stepwise feature selection was applied to all identified peptides. Given the cohort size of 23 individuals, the number of predictors was limited to four to reduce overfitting risk. Candidate peptides were preselected according to single-feature model area under the curve (AUC), optimal combinations were chosen by Akaike information criterion (AIC), and the final model was evaluated by leave-one-out cross-validation (LOOCV).

All analyses and figure generation were carried out in R using limma, mixOmics, dplyr, tidyr, pROC, boot, ggplot2, ComplexHeatmap, grid, gridExtra, ggrepel, and RColorBrewer.

## 3. Results and Discussion

The study included 8 healthy donors and 15 patients with MetS (Table 1; Supplementary Table S1, Sheet S1). The MetS group was further stratified according to glycemic status into normoglycemia, prediabetes, and T2DM (n = 5 in each group). The baseline characteristics of the study participants are presented in Table 1.

**Figure 1:**
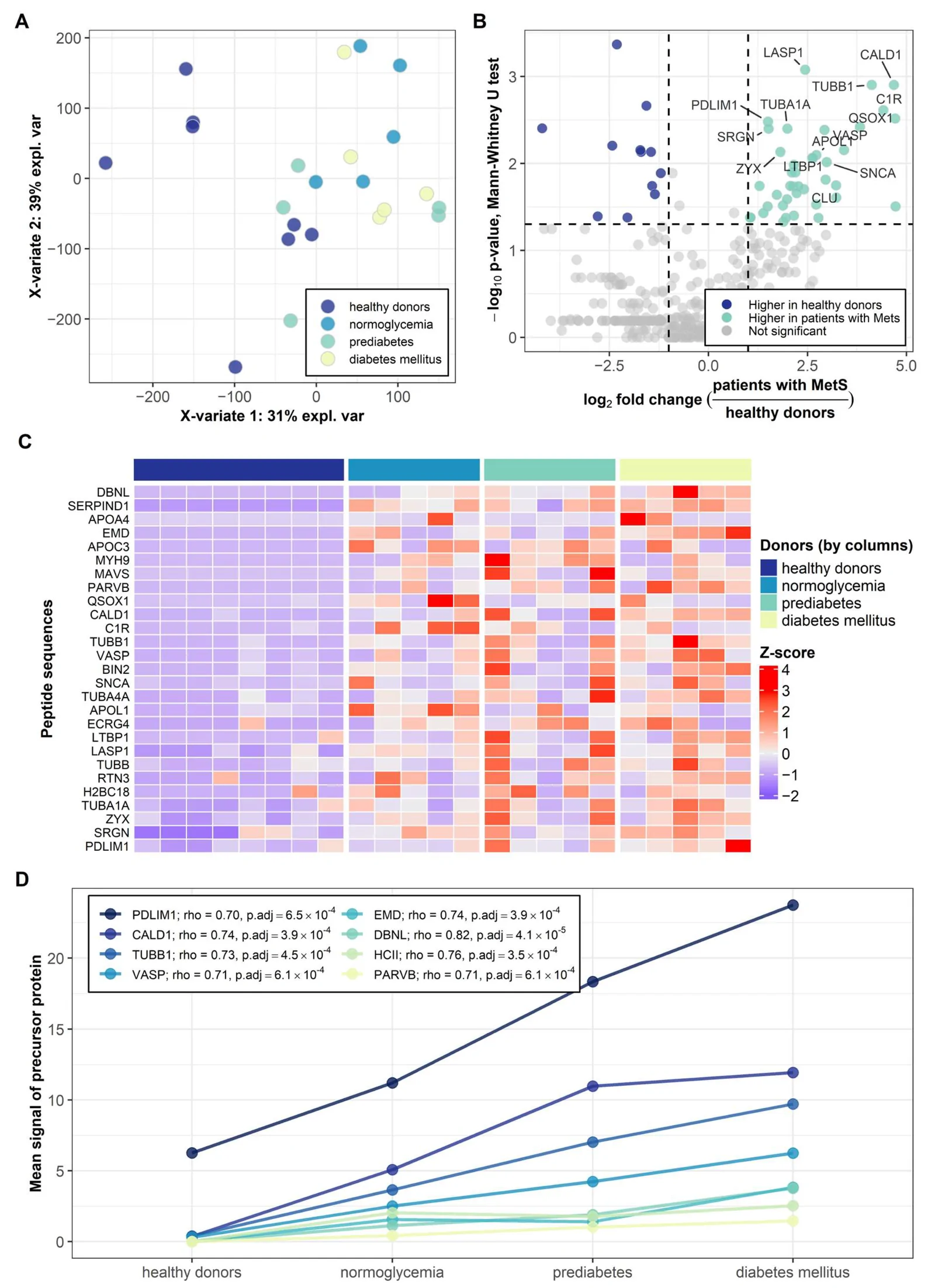
Serum from patients with MetS contains increased levels of peptides derived from disease-associated precursor proteins. (**A**) Partial least squares-discriminant analysis of the global precursor-protein profile across healthy donors and patients with MetS. (**B**) Volcano plot of precursor proteins differing in cumulative peptide signal between healthy donors and patients with metabolic syndrome. (**C**) Heatmap of 27 precursor proteins across all donors with annotation of donor groups. (**D**) Precursor proteins whose aggregate peptide signal was positively associated with progression from healthy status to T2DM.

LC-MS/MS analysis identified 6754 unique peptides corresponding to 1820 precursor proteins (Supplementary Table S1, Sheets S2 and S3). Partial least squares-discriminant analysis (PLS-DA) revealed clear separation between healthy donors and patients with MetS, with the most pronounced displacement observed in the diabetes mellitus subgroup, indicating the presence of a stage-related disease signal (Figure 1A). These findings motivated a stepwise analysis of potential peptide markers by (1) comparing healthy donors with all patients with MetS and (2) subsequently focusing on the comparison between healthy donors and patients with prediabetes.

According to our analytical workflow, differences were first assessed at the precursor-protein level and then refined at the peptide level. This design increased the statistical interpretability of the data and prioritized peptide candidates supported by coordinated changes in their parent protein-derived peptide pools.

### 3.1. Serum from Patients with Metabolic Syndrome Contains Overrepresented Peptides Derived from Precursor Proteins Previously Associated with the Disease

Differential analysis between healthy donors and patients with MetS at the precursor-protein level was performed for proteins whose peptides were identified in at least 10 patients with MetS (Figure 1B). Among precursor proteins showing increased cumulative peptide signal in MetS (log2FC ≥ 1 and *p*.adj ≤ 0.05), several were functionally linked to lipid or glucose metabolism (Figure 1C; Supplementary Table S2, Sheet S1). In particular, apolipoprotein A-IV (APOA4; not detected in healthy donors, *p*.adj = 0.028), which is involved in lipid transport, reverse cholesterol transport, and the regulation of glucose homeostasis [28], may be regarded as one of the most biologically plausible markers of cardiometabolic dysfunction [29]. Similarly, apolipoprotein L1 (APOL1; log2FC = 2.7, *p*.adj = 0.035), which has been associated with insulin resistance and altered lipid metabolism [30], is also likely linked to cardiometabolic abnormalities. In addition, alpha-synuclein (SNCA; log2FC = 3.0, *p*.adj = 0.038), previously described as contributing to glucose uptake and utilization [31] and associated with insulin resistance [32], further points to the involvement of glucose metabolism-related regulatory mechanisms.

A distinct group was represented by precursor proteins linking metabolic abnormalities to inflammatory and vascular mechanisms. In particular, vasodilator-stimulated phosphoprotein (VASP; log2FC = 3.8, *p*.adj = 0.028), which is involved in NO/cGMP signaling and has been implicated in protection against obesity-associated hepatic insulin resistance [33], points to the contribution of vascular–metabolic pathways to the observed differences. A similar pattern was observed for complement C1r subcomponent (C1R; log2FC = 4.4, *p*.adj = 0.028), a marker of complement system activation in diabetic kidney disease [34], further supporting a link between metabolic dysregulation and inflammatory responses. Likewise, mitochondrial antiviral-signaling protein (MAVS; not detected in healthy donors, *p*.adj = 0.028) may also be of interest, as it has been reported to coordinate innate immune signaling and glucose metabolism [35].

Another logical group comprised precursor proteins primarily reflecting tissue remodeling and organ complications of diabetes. For example, caldesmon 1 (CALD1; log2FC = 4.7, *p*.adj = 0.024), which has been associated with diabetic nephropathy [36], and latent transforming growth factor beta-binding protein 1 (LTBP1; log2FC = 2.6, *p*.adj = 0.035), linked to extracellular matrix remodeling and the progression of diabetic kidney disease [37], point to a fibrotic and vascular–tissue component of metabolic disturbances. Similarly, LIM and SH3 domain protein 1 (LASP1; log2FC = 2.4, *p*.adj = 0.024), involved in the profibrotic response to high glucose in renal tissue [38], further supports the presence of diabetes-associated remodeling. In turn, PDZ and LIM domain protein 1 (PDLIM1; log2FC = 1.5, *p*.adj = 0.028) and serglycin (SRGN; log2FC = 1.5, *p*.adj = 0.028) may likewise represent candidate markers of diabetic vascular complications, including diabetic retinopathy [39,40].

### 3.2. Precursor Proteins Whose Cumulative Peptide Signals Were Associated with Glycemic Stage

Spearman correlation analysis identified precursor proteins whose cumulative peptide signals increased progressively from healthy donors to patients with normoglycemia, prediabetes, and diabetes mellitus (Figure 1D; Supplementary Table S2, Sheet S1). Among the most prominent stage-associated precursor proteins were drebrin-like protein (DBNL), heparin cofactor II (HCII), emerin (EMD), CALD1, tubulin beta-1 chain (TUBB1), VASP, beta-parvin (PARVB), and PDLIM1. For most of these proteins, the increase became apparent already at the early stages of dysglycemia and, in several cases, was further accentuated in patients with diabetes mellitus.

HCII was characterized by particularly pronounced dynamics and showed one of the strongest correlations: the cumulative signal of all identified peptides derived from this precursor protein was strongly associated with the degree of glucose metabolism impairment (rho = 0.76, *p*.adj = 3.5 × 10^−4^). Thus, the HCII-related signal may be regarded as one of the most prominent potential markers linked to metabolic disturbances, in agreement with previous reports [41,42,43]. Among the other notable proteins, PARVB (rho = 0.71, *p*.adj = 6.1 × 10^−4^) is of particular interest, as its association with metabolic abnormalities may plausibly be related to its reported links to the development and progression of metabolic dysfunction-associated liver disease [44,45]. TUBB1 (rho = 0.73, *p*.adj = 4.5 × 10^−4^) is also noteworthy, as it may reflect the vascular and thrombotic component of cardiometabolic risk [46], which is often exacerbated in insulin resistance and T2DM [47,48].

### 3.3. Analysis of Only a Subset of the Identified Peptides Revealed Potential Peptide Markers for MetS

**Figure 2:**
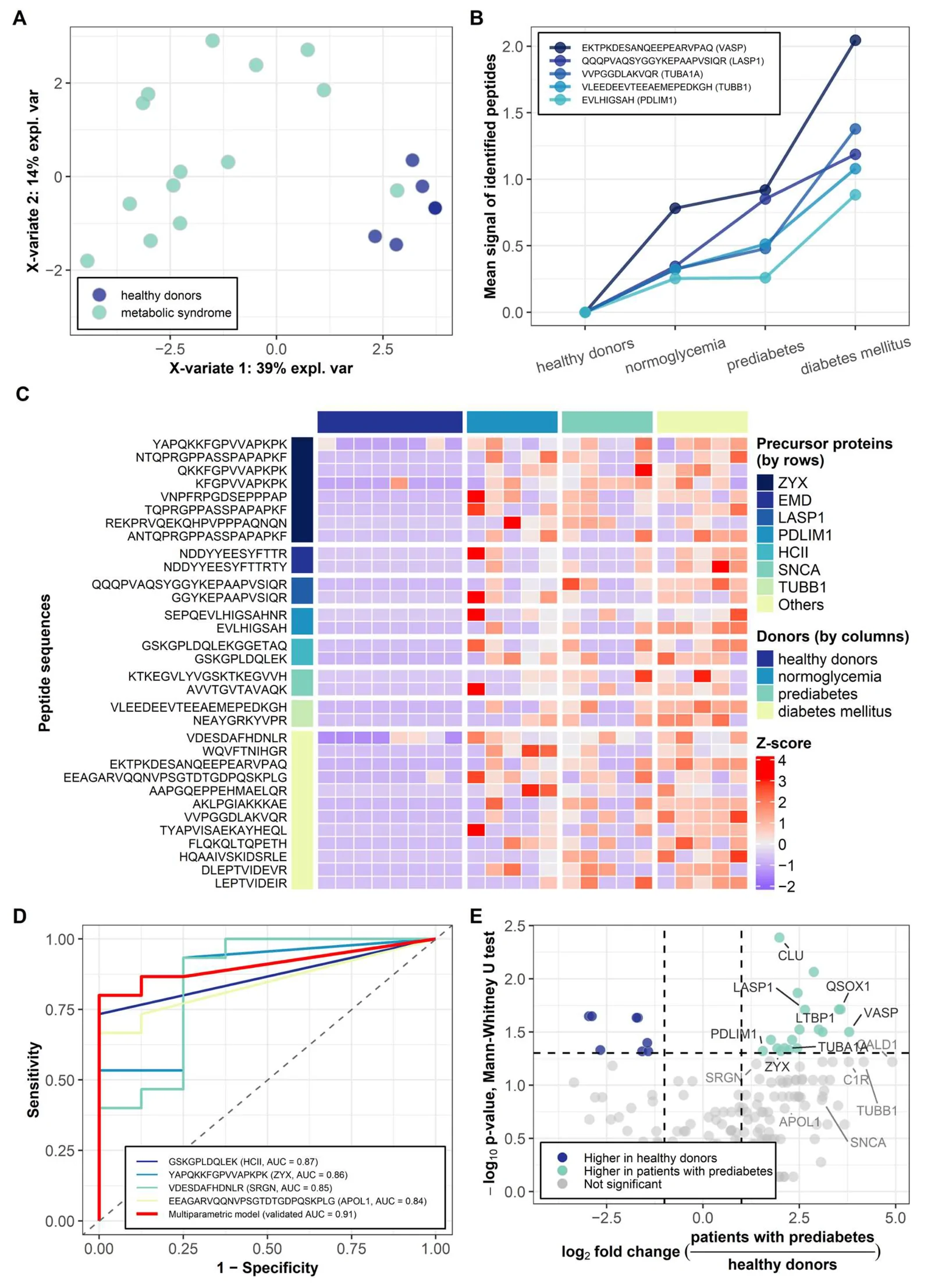
The analytical workflow identified 32 candidate peptide markers of metabolic syndrome, including peptides potentially informative at early dysglycemic stages. (**A**) Partial least squares-discriminant analysis of 32 differentially overrepresented peptides in patients with metabolic syndrome. (**B**) Top peptides whose PSMs were positively associated with progression across metabolic stages. (**C**) Heatmap of the 32 candidate peptide markers across all donors with precursor protein and donor group annotation. (**D**) Receiver operating characteristic curves for individual peptide models and for the four-peptide multivariate logistic regression model. (**E**) Volcano plot of precursor proteins differing in cumulative peptide signal between healthy donors and patients with prediabetes.

After restricting analysis to peptides derived from the previously selected precursor proteins, 32 peptides remained that were identified in at least eight patients with MetS and met the threshold criteria of log2FC ≥ 1 and *p*.adj ≤ 0.05. PLS-DA based on these peptides provided clearer separation between healthy donors and patients (Figure 2A) than the global precursor-protein profile (Figure 1A), indicating that focused peptide-level analysis may sharpen disease discrimination.

Among the 32 peptides with the most pronounced signal in the MetS group (Figure 2C; Supplementary Table S2, Sheet S2) were EEAGARVQQNVPSGTDTGDPQSKPLG (log2FC = 3.1, *p*.adj = 0.027) derived from APOL1 and AAPGQEPPEHMAELQR (not detected in healthy donors, *p*.adj = 0.035), corresponding to sulfhydryl oxidase 1 (QSOX1). The QSOX1-related signal may reflect redox-dependent vascular and tissue remodeling accompanying cardiometabolic disturbances in these patients [49,50]. Particular attention should be paid to precursor proteins represented by multiple significant peptide fragments (Figure 2C). Such reproducibility strengthens the likelihood that these proteins are genuinely linked to disease-associated serum peptidome remodeling. In this respect, zyxin (ZYX) was especially notable, as differential analysis identified eight peptides derived from this precursor protein. Similarly, PDLIM1, HCII, TUBB1, LASP1, EMD, and SNCA were each represented by two differentially significant identified peptides.

Spearman correlation analysis identified five peptides showing stage-associated changes across glycemic progression (Figure 2B; Supplementary Table S2, Sheet S2). The most pronounced pattern was observed for peptides derived from VASP, LASP1, tubulin alpha chain (TUBA1A), TUBB1, and PDLIM1.

Among the identified peptides, the TUBA1A-derived peptide VVPGGDLAKVQR showed a particularly strong and consistent stage-associated pattern (rho = 0.79, *p*.adj = 1.9 × 10^−4^). Because TUBA1A is a structural component of microtubules, the increase in this signal may reflect not merely an isolated change in a single protein, but rather a broader process of cytoskeletal remodeling accompanying the progression of glucose metabolism disturbances. Microtubules are involved in the regulation of insulin secretion in β-cells and, in platelets, play a key role in maintaining cell shape, cytoskeletal reorganization, and activation responses [51]. This interpretation is further supported by similar stage-associated changes observed for TUBB1, VASP, LASP1, and PDLIM1, proteins functionally linked to microtubules, actin-associated structures, cell adhesion, and platelet activation.

### 3.4. A Multivariate Logistic Regression Model Identified a Panel of Four Peptides That Potentially Enables the Differentiation of Healthy Donors from Patients with MetS

As a complementary classification approach, multivariate logistic regression was constructed to discriminate between healthy donors and patients with MetS. At the first stage, individual logistic regression models were built for each peptide, and the 10 peptides with the highest model AUC values were selected: WQVFTNIHGR (C1R, AUC = 0.87), EKTPKDESANQEEPEARVPAQ (VASP, AUC = 0.87), VNPFRPGDSEPPPAP (ZYX, AUC = 0.87), QQQPVAQSYGGYKEPAAPVSIQR (LASP1, AUC = 0.87), GSKGPLDQLEK (HCII, AUC = 0.87), YAPQKKFGPVVAPKPK (ZYX, AUC = 0.86), VDESDAFHDNLR (SRGN, AUC = 0.85), EEAGARVQQNVPSGTDTGDPQSKPLG (APOL1, AUC = 0.84), SEPQEVLHIGSAHNR (PDLIM1, AUC = 0.83), QKKFGPVVAPKPK (ZYX, AUC = 0.83). Following Akaike information criterion (AIC)-based selection, the final multiparametric model included the following four peptides: GSKGPLDQLEK (HCII), YAPQKKFGPVVAPKPK (ZYX), VDESDAFHDNLR (SRGN), and EEAGARVQQNVPSGTDTGDPQSKPLG (APOL1). The validated AUC of the multivariate model, calculated using leave-one-out cross-validation (LOOCV), was 0.91, indicating good discrimination between healthy donors and patients with MetS (Figure 2D). A model based on only four peptides is convenient for interpretation and further validation in independent cohorts.

The multivariate model included peptide fragments derived from four different proteins associated with distinct biological contexts. This potentially suggests that the discriminative signal is driven not by a single dominant protein, but rather by a combination of peptide-level features originating from different molecular sources. These peptides had previously been identified as potential markers in the comparative analysis of peptides detected in healthy donors and patients with MetS. The multivariate model demonstrated stronger discriminatory performance than the individual peptides considered separately, though they both warrant further investigation.

### 3.5. Differential Analysis of Identified Peptides Enabled the Identification of Potential Peptide Markers for Prediabetes

As some interesting stage-associated trends were observed for the identified peptides and their precursor proteins, a separate comparative analysis of healthy donors and patients with prediabetes was performed to search for possible earlier biomarker candidates (Figure 2E). For this analysis, only those proteins were included whose peptides were identified in at least four of the five patients with prediabetes. Candidate selection was then based on the threshold of log2FC ≥ 1 and *p*.adj ≤ 0.1 (Supplementary Table S3, Sheet S1), since applying a more stringent threshold (*p*.adj ≤ 0.05) yielded few candidates for meaningful interpretation.

Among the precursor proteins identified exclusively in the separate comparison of healthy donors and patients with prediabetes were clusterin (CLU; log2FC = 2.0, *p*.adj = 0.071), pituitary tumor-transforming gene 1 protein-interacting protein (PTTG1IP; not detected in healthy donors, *p*.adj = 0.071), and tight junction protein 2 (TJP2; not detected in healthy donors, *p*.adj = 0.071). According to published data, CLU is regarded as a stress-associated secreted protein linked to insulin resistance, inflammation, and cardiometabolic disturbances [52], making it a biologically plausible candidate marker of early alterations in prediabetes.

Peptide-level analysis was then performed for the fragments of the selected precursor proteins. Peptides were set to be detected in at least three patients with prediabetes and satisfy the criteria of log2FC ≥ 1 and *p*.adj ≤ 0.05 (Supplementary Table S3, Sheet S2). Among the peptides significantly increased in patients with prediabetes, the most notable were AQQAADKYLYVD (not detected in healthy donors, *p*.adj = 0.010), a fragment of myosin-9 (MYH9) identified in all five patients with prediabetes, as well as KEPRYQEEPPAPQPK (TJP2; not detected in healthy donors, *p*.adj = 0.017) and EENPYARFENN (PTTG1IP; not detected in healthy donors, *p*.adj = 0.017), both detected in four patients with prediabetes.

Despite the identification of these candidate peptides, the overall alterations in the prediabetes subgroup were less pronounced and less consistent than those observed in patients with normoglycemic MetS. This pattern may reflect biological heterogeneity during the early stages of glycemic impairment, together with the limited statistical power of the small subgroups and technical variability inherent to discovery-based serum peptidomics.

### 3.6. According to the Identified Peptides and Their Precursor Proteins MetS Is a Condition Potentially Linked to Systemic Acceleration of Biological Aging

Among the 273 conserved plasma proteins associated with aging, the dominant pathways involve inflammation, complement activation, extracellular matrix (ECM) remodeling, and metabolic regulation. The expanded hallmarks-of-aging framework [53], together with the concept of the inflammaging–metaflammation continuum [54], suggests understanding of how MetS-associated proteins may act as molecular indicators of accelerated biological aging. A potential connection between MetS and aging is reflected in the process of cellular senescence. Hyperinsulinemia can promote the onset of senescence, while senescent cells in turn may exacerbate insulin resistance, creating a self-reinforcing cycle [55]. In parallel, individuals with MetS show increased DNA and RNA damage [56], further suggesting the idea of premature cellular decline.

An example is the mitochondrial antiviral-signaling protein MAVS. In senescent cells, mitochondrial dysfunction may permit double-stranded RNA to leak into the cytosol, activating the RIG-I/MDA5/MAVS pathway [57]. This inflammatory dimension also extends to complement activation and immune-cell regulation. C1R is involved in the classical complement cascade, a system known to become increasingly activated with age [58]. Likewise, SRGN contributes to both neuroinflammation and adipose tissue inflammation by promoting proinflammatory signaling and macrophage polarization [59,60]. These processes are intensified in both aging and obesity, which warrants further investigation as a shared inflammatory background.

Another major point of convergence may be vascular aging. Loss of CALD1 during smooth muscle cell phenotypic switching contributes to arterial stiffening [61], while SERPIND1 activity decreases with age and is inversely associated with atherosclerotic burden [62]. ECM dysregulation, now recognized as an emerging hallmark of aging [63], is also strongly altered in MetS. Increased LTBP1 may reflect altered TGF-β regulation linked to fibrotic remodeling [64,65]. QSOX1, although important for tissue homeostasis through oxidative protein folding, may also contribute to oxidative stress through its production of hydrogen peroxide [66,67].

Overall, the identified peptides and their precursor proteins collectively reflect multiple core hallmarks of aging, including chronic inflammation, vascular dysfunction, impaired tissue remodeling, loss of mechanical resilience, and organ-specific decline. Their coordinated alteration suggests that MetS is not only a state of metabolic imbalance but also a condition potentially linked to systemic acceleration of biological aging.

### 3.7. Features of the Analytical Workflow and Limitations of the Obtained Results

The analytical workflow used in this study was shaped by the intrinsic variability of the blood serum peptidome [68,69,70], together with the pilot nature of the study and the small cohort size. Under these conditions, direct differential testing of all identified peptides with multiple-testing correction would likely have yielded few significant results, limiting biological interpretation. The stepwise strategy therefore served to reduce the multiple-comparison burden and to focus downstream analysis on signals already supported at the precursor-protein level.

It is important to emphasize that peptidomics study assesses precursor proteins indirectly through their identified peptide fragments, that is, through the degradome. Accordingly, increased representation of fragments from a given protein may reflect higher protein abundance, enhanced proteolytic processing, or a combination of both. These possibilities should be addressed in future validation studies. Within the framework of this study, the observed degradome was particularly informative when multiple fragments derived from the same precursor protein were identified simultaneously. Such a protein is likely sensitive to metabolic disturbances and may serve as a source of potential disease biomarkers circulating in the bloodstream.

The detection of peptide fragments derived from intracellular, mitochondrial, nuclear, and cytoskeletal proteins should not be interpreted as evidence that the corresponding intact proteins act as actively secreted circulating regulators. Previous plasma proteomic studies have shown that a substantial proportion of medium- and low-abundance plasma proteins are intracellular or tissue-leakage proteins originating from normal cell turnover, damaged tissues, or dying cells [71,72,73]. Accordingly, the increased representation of fragments derived from MAVS, CALD1, EMD, tubulins, and other intracellular proteins may reflect enhanced cellular injury, apoptosis, or systemic tissue turnover associated with MetS, with blood serving as a compartment in which molecular signals released from different tissues can be detected.

Small cohort size limited the statistical power of the subgroup analyses and increases the possibility that some of the observed associations are cohort-specific. Although the four-peptide logistic regression model was evaluated using leave-one-out cross-validation across the full cohort, feature selection and model assessment were performed within the same small dataset. Therefore, the reported ROC AUC may be unstable or overly optimistic, and the proposed peptide panel should be regarded as strictly preliminary.

The prediabetes analysis was additionally limited by the use of a relaxed significance threshold of *p*.adj ≤ 0.1 at the precursor-protein level. Although this threshold was applied to retain potentially informative candidates in an exploratory setting, it increases the likelihood of false-positive findings. Accordingly, all identified candidates require validation in larger independent cohorts. As a next step, we plan to perform targeted quantitative validation using multiple reaction monitoring and to include the maximum feasible number of candidate peptides identified in the present discovery analysis.

An additional limitation is the use of blood serum obtained after coagulation for up to 1 h without immediate addition of protease inhibitors. Proteolytic activity during coagulation and pre-analytical processing may have generated or further modified some peptide fragments ex vivo; therefore, not all identified peptides can be unequivocally regarded as native circulating species [74,75]. Nevertheless, all samples were collected and processed according to the same standardized protocol, reducing the likelihood that systematic pre-analytical differences alone account for the observed separation between the study groups, although this possibility cannot be fully excluded. Serum is widely used alongside plasma in proteomic and peptidomic biomarker studies because it is routinely available in clinical laboratories and provides a reproducible, though partly distinct, molecular profile [76,77].

Some differences in mean age and medication use were present between the study groups. Although the exclusion of statin and glucocorticoid use and the absence of prior antidiabetic therapy in patients with newly diagnosed diabetes reduced the influence of selected medications, residual demographic and treatment-related confounding cannot be excluded. In addition, the cross-sectional design and absence of longitudinal follow-up did not allow assessment of within-patient changes in peptide profiles over time or after treatment initiation. These limitations should be considered when interpreting the findings of this pilot study and should be addressed in larger, prospectively recruited cohorts with closer matching of demographic characteristics, detailed medication stratification, and longitudinal sampling.

## 4. Conclusions

In this study, a comprehensive analysis of peptides circulating in the bloodstream of 8 healthy donors and 15 patients with MetS identified 32 potential peptide markers overrepresented in MetS, taking into account both the level of precursor proteins (Figure 1C) and the level of the identified peptides themselves (Figure 2C). Collectively, these findings not only enabled the identification of potential peptide markers associated with MetS and impaired glucose metabolism, but also supported the development of a statistically more robust multivariate logistic regression model based on four candidate markers, yielding a validated AUC of 0.91 (Figure 2D). Combining several peptide-level features derived from different molecular sources makes it possible to better capture the multifactorial nature of MetS. In addition, the small size of the panel makes it practically convenient for subsequent validation using targeted approaches.

Moreover, stage-associated changes were observed across the transition from healthy donors to normoglycemia, prediabetes, and T2DM: 8 precursor proteins were identified at the protein level (Figure 1D), and 5 corresponding peptide fragments were identified at the peptide level (Figure 2B). This pattern potentially suggests that the observed changes reflect not random intergroup differences, but rather coordinated functional alterations. Of particular importance is the fact that some signals became detectable already at the prediabetes stage, that is, potentially before the development of more pronounced disturbances in glucose metabolism, and may be exploited in the search for early biomarkers.

The obtained data show that, on the one hand, the identified candidate peptides are derived from precursor proteins that fit well into already established pathophysiological mechanisms of MetS, including disturbances in lipid and glucose metabolism (APOA4 [22,23], APOL1 [24], SNCA [25,26], VASP [27], MAVS [29], PARVB [38,39], and HCII [35,36,37]), inflammation (C1R [28], MAVS [29], and SRGN [34]), vascular dysfunction (VASP [27], C1R [28], CALD1 [30], LTBP1 [31], LASP1 [32], PDLIM1 [33], SRGN [34], TUBB1 [40,41,42], and QSOX1 [43,44]), coagulation-related shifts (HCII [35,36,37] and TUBB1 [40,41,42]), and tissue remodeling (CALD1 [30], LTBP1 [31], LASP1 [32], PDLIM1 [33], PARVB [38,39], TUBB1 [40,41,42], and QSOX1 [43,44]). On the other hand, some of the detected peptides may represent novel candidate markers that have not previously been considered in the context of MetS, including proteins such as drebrin-like protein (DBNL), emerin (EMD), and zyxin (ZYX). Thus, our analytical workflow not only confirmed previously reported associations, but also expanded the range of molecular candidates that warrant further investigation.

Given the pilot nature of the present study, the limited cohort size, and the absence of an independent validation cohort, the identified peptide candidates and the four-peptide classification model should be regarded as preliminary and hypothesis-generating. In particular, the reported discriminatory performance requires confirmation in larger independent cohorts, while the candidates identified in the small prediabetes subgroup require cautious interpretation because of the relaxed statistical threshold used in this exploratory analysis.

Since the candidate markers were identified using a discovery-based approach, we consider the next stage of this work to be a transition to targeted quantitative analysis using, for example, a multiple reaction monitoring (MRM) approach. We plan to include the maximum feasible number of MetS-, glycemic-stage-, and prediabetes-associated candidate peptides identified in the present study, rather than validating only the four peptides selected for the preliminary multivariate model. Candidate prioritization will be based on detection frequency, effect size, statistical significance, association with glycemic stage, biological relevance of the precursor protein, and analytical suitability for targeted mass spectrometry. The use of targeted LC-MS/MS would make it possible to assess the reproducibility of the identified peptide signals in larger and independent cohorts, improve the accuracy and sensitivity of the measurements, and move from the semi-quantitative recording of identifications to a more rigorous quantitative assessment of candidate marker levels [78,79]. This approach is particularly well suited to small multivariate panels and may represent a necessary step toward the clinical validation of the proposed four-peptide multivariate logistic regression model.

## Figures and Tables

**Table 1:** Baseline characteristics of the study participants.

Variables	Healthy Donors	Patients with MetS
Age, years; mean ± SD	39.9 ± 6.8	52.2 ± 8.9
Gender; male/female	4/4	9/6
BMI; mean ± SD	24.1 ± 1.3	31.1 ± 6.6
Antihypertensive medications	0 of 8	8 of 15
WC, cm	79.2 ± 14.2	105.9 ± 14.0
TG, mmol/L	1.1 ± 0.6	1.3 ± 0.4
HDL-C, mmol/L	1.3 ± 0.3	1.2 ± 0.5
Fasting plasma glucose, mmol/L	5.1 ± 0.6	5.4 ± 0.5
HbA1c, %	5.5 ± 0.3	5.6 ± 0.7

## Data Availability

The mass spectrometry peptidomics data have been deposited to the ProteomeXchange Consortium via the PRIDE partner repository with the dataset identifier PXD076281 and 10.6019/PXD076281.

## Supplementary Materials

The following supporting information can be downloaded at https://www.mdpi.com/article/10.3390/jpm16080425/s1, Supplementary Table S1: Summary tables of LC-MS/MS serum peptide profiling of healthy donors and patients with metabolic syndrome. Sheet S1—Donor descriptions and identification summary; Sheet S2—Identified peptide sequences by donor; Sheet S3—Precursor proteins by donor. Supplementary Table S2: Differential analysis of LC-MS/MS serum peptide profiling of healthy donors and patients with metabolic syndrome. Sheet S1—Differential analysis of precursor proteins based on total corresponding peptide PSMs; Sheet S2—Differential analysis of identified peptides. Supplementary Table S3: Differential analysis of LC-MS/MS serum peptide profiling of healthy donors and patients with prediabetes. Sheet S1—Differential analysis of precursor proteins based on total corresponding peptide PSMs; Sheet S2—Differential analysis of identified peptides.

## References

[1] Després J.-P., Lemieux I. (2006). Abdominal Obesity and Metabolic Syndrome. Nature.

[2] Chaudhari A., Prabhu P., Tayade M., Bhangale K. (2025). Lipid Profile Abnormalities in Metabolic Syndrome Patients: A Comparative Cross-Sectional Study. Eur. J. Cardiovasc. Med..

[3] Kumar Karn S., Zhao F., Xu Y., Zhang Y., Zeng C., Shaikh I.I., Singh S., Feng Y. (2025). Metabolic Syndrome with Mortality and Major Adverse Cardiovascular Events in an Elderly Population. Front. Endocrinol..

[4] Van Jaarsveldt C., Jabari T., Zwarts E., Färber S., Sikuza Y., Schilling H., Pauw S., Klein E., Van Rooyen C., Joubert G. (2024). Prevalence of Metabolic Syndrome among Adults Treated at a District Hospital Outpatient Department. S. Afr. Fam. Pr..

[5] James M., Varghese T.P., Sharma R., Chand S. (2020). Association between Metabolic Syndrome and Diabetes Mellitus according to International Diabetic Federation and National Cholesterol Education Program Adult Treatment Panel III Criteria: A Cross-Sectional Study. J. Diabetes Metab. Disord..

[6] Li W., Chen D., Peng Y., Lu Z., Kwan M.-P., Tse L.A. (2023). Association between Metabolic Syndrome and Mortality: Prospective Cohort Study. JMIR Public Health Surveill..

[7] Onesi S.O., Ignatius U.E. (2014). Metabolic Syndrome: Performance of Five Different Diagnostic Criterias. Indian J. Endocrinol. Metab..

[8] Guo F., Moellering D.R., Garvey W.T. (2014). Use of HbA1c for Diagnoses of Diabetes and Prediabetes: Comparison with Diagnoses Based on Fasting and 2-Hr Glucose Values and Effects of Gender, Race, and Age. Metab. Syndr. Relat. Disord..

[9] Aekplakorn W., Tantayotai V., Numsangkul S., Sripho W., Tatsato N., Burapasiriwat T., Pipatsart R., Sansom P., Luckanajantachote P., Chawarokorn P. (2015). Detecting Prediabetes and Diabetes: Agreement between Fasting Plasma Glucose and Oral Glucose Tolerance Test in Thai Adults. J. Diabetes Res..

[10] Overgaard K.S., Andersen T.R., Mohamed R.A., Heinsen L.J., Binderup H.G., Möller S., Auscher S., Lambrechtsen J., Egstrup K. (2023). Can Prediabetes Diagnosed Using HemoglobinA1c or Oral Glucose Tolerance Test Predict Presence and Severity of Coronary Artery Disease in Symptomatic Patients?. Diab. Vasc. Dis. Res..

[11] Mulla I.G., Anjankar A., Pratinidhi S., Agrawal S.V., Gundpatil D., Lambe S.D. (2024). Prediabetes: A Benign Intermediate Stage or a Risk Factor in Itself?. Cureus.

[12] Zhang Z., Svensson K.J. (2025). Discovery of Peptides as Key Regulators of Metabolic and Cardiovascular Crosstalk. Cell Rep..

[13] Elhadad M.A., Wilson R., Zaghlool S.B., Huth C., Gieger C., Grallert H., Graumann J., Rathmann W., Koenig W., Sinner M.F. (2021). Metabolic Syndrome and the Plasma Proteome: From Association to Causation. Cardiovasc. Diabetol..

[14] Yu Y., Jiang X., Pei B., Zhou J., Long Z., Cao Y., Ye J., Gao Y., Xie K., Yuan H. (2026). Proteome Profiling Reveals Early Diagnostic Biomarker Candidates for Colorectal Cancer. BMC Cancer.

[15] Zhu Y. (2024). Plasma/Serum Proteomics Based on Mass Spectrometry. Protein Pept. Lett..

[16] Li L., Wu J., Lyon C.J., Jiang L., Hu T.Y. (2023). Clinical Peptidomics: Advances in Instrumentation, Analyses, and Applications. BME Front..

[17] Repetto O., Vettori R., Steffan A., Cannizzaro R., De Re V. (2023). Circulating Proteins as Diagnostic Markers in Gastric Cancer. Int. J. Mol. Sci..

[18] Muqaku B., Dorst J., Wiesenfarth M., Otto M., Ludolph A.C., Oeckl P. (2025). Peptidomic Analysis of CSF Reveals New Biomarker Candidates for Amyotrophic Lateral Sclerosis. EMBO Mol. Med..

[19] Stewart R.A.H., Robledo K.P., Tonkin A.M., Keech A., Kritharides L., Marschner I., Janus E., Thompson P.L., Watts G.F., Zeller T. (2024). Plasma Protein Biomarkers and Long-Term Cardiovascular Mortality Risk in Patients with Chronic Coronary Heart Disease. J. Am. Heart Assoc..

[20] Arapidi G.P., Urban A.S., Osetrova M.S., Shender V.O., Butenko I.O., Bukato O.N., Kuznetsov A.A., Saveleva T.M., Nos G.A., Ivanova O.M. (2024). Non-Human Peptides Revealed in Blood Reflect the Composition of Intestinal Microbiota. BMC Biol..

[21] Latosinska A., Frantzi M., Siwy J. (2024). Peptides as “Better Biomarkers”? Value, Challenges, and Potential Solutions to Facilitate Implementation. Mass Spectrom. Rev..

[22] Tirumalai R.S., Chan K.C., Prieto D.A., Issaq H.J., Conrads T.P., Veenstra T.D. (2003). Characterization of the Low Molecular Weight Human Serum Proteome. Mol. Cell. Proteom..

[23] Lopez M.F., Mikulskis A., Kuzdzal S., Golenko E., Petricoin E.F., Liotta L.A., Patton W.F., Whiteley G.R., Rosenblatt K., Gurnani P. (2007). A Novel, High-Throughput Workflow for Discovery and Identification of Serum Carrier Protein-Bound Peptide Biomarker Candidates in Ovarian Cancer Samples. Clin. Chem..

[24] Lowenthal M.S., Mehta A.I., Frogale K., Bandle R.W., Araujo R.P., Hood B.L., Veenstra T.D., Conrads T.P., Goldsmith P., Fishman D. (2005). Analysis of Albumin-Associated Peptides and Proteins from Ovarian Cancer Patients. Clin. Chem..

[25] Rifai N., Gillette M.A., Carr S.A. (2006). Protein Biomarker Discovery and Validation: The Long and Uncertain Path to Clinical Utility. Nat. Biotechnol..

[26] Bader J.M., Albrecht V., Mann M. (2023). MS-Based Proteomics of Body Fluids: The End of the Beginning. Mol. Cell. Proteom..

[27] Vizcaíno J.A., Csordas A., del-Toro N., Dianes J.A., Griss J., Lavidas I., Mayer G., Perez-Riverol Y., Reisinger F., Ternent T. (2016). 2016 Update of the PRIDE Database and Its Related Tools. Nucleic Acids Res..

[28] Qu J., Ko C.-W., Tso P., Bhargava A. (2019). Apolipoprotein A-IV: A Multifunctional Protein Involved in Protection against Atherosclerosis and Diabetes. Cells.

[29] Li L.-Y., Chen S., Wang Y.-X., Ji R., Ding F.-H., Wang X.-Q., Chen Q.-J., Lu L., Dai Y. (2022). Serum Apolipoprotein A-IV Levels Are Associated with Flow-Mediated Dilation in Patients with Type 2 Diabetes Mellitus. BMC Cardiovasc. Disord..

[30] Mukamal K.J., Tremaglio J., Friedman D.J., Ix J.H., Kuller L.H., Tracy R.P., Pollak M.R. (2016). APOL1 Genotype, Kidney and Cardiovascular Disease, and Death in Older Adults. Arterioscler. Thromb. Vasc. Biol..

[31] Rodriguez-Araujo G., Nakagami H., Hayashi H., Mori M., Shiuchi T., Minokoshi Y., Nakaoka Y., Takami Y., Komuro I., Morishita R. (2013). Alpha-Synuclein Elicits Glucose Uptake and Utilization in Adipocytes through the Gab1/PI3K/Akt Transduction Pathway. Cell. Mol. Life Sci..

[32] Rodriguez-Araujo G., Nakagami H., Takami Y., Katsuya T., Akasaka H., Saitoh S., Shimamoto K., Morishita R., Rakugi H., Kaneda Y. (2015). Low Alpha-Synuclein Levels in the Blood Are Associated with Insulin Resistance. Sci. Rep..

[33] Tateya S., Rizzo N.O., Handa P., Cheng A.M., Morgan-Stevenson V., Daum G., Clowes A.W., Morton G.J., Schwartz M.W., Kim F. (2011). Endothelial NO/cGMP/VASP Signaling Attenuates Kupffer Cell Activation and Hepatic Insulin Resistance Induced by High-Fat Feeding. Diabetes.

[34] Yang Y., Zhang Y., Li Y., Zhou X., Honda K., Kang D., Wang M., Yang J.-H., Xia Z., Wei Y. (2025). Complement Classical and Alternative Pathway Activation Contributes to Diabetic Kidney Disease Progression: A Glomerular Proteomics on Kidney Biopsies. Sci. Rep..

[35] He Q.-Q., Huang Y., Nie L., Ren S., Xu G., Deng F., Cheng Z., Zuo Q., Zhang L., Cai H. (2023). MAVS Integrates Glucose Metabolism and RIG-I-like Receptor Signaling. Nat. Commun..

[36] Millioni R., Iori E., Lenzini L., Puricelli L., Caroccia B., Arrigoni G., Rossi G.P., Tessari P. (2011). Caldesmon over-Expression in Type 1 Diabetic Nephropathy. J. Diabetes Complicat..

[37] Hu S., Tian M., Hu W., Yao L., Tang Y., Shen W., He Q., Xu J., Yao H., Ji L. (2025). The Cuproptosis-Related Gene ITGB6 and LTBP1 May Be Associated with Diabetic Kidney Disease Progression and Immune Cell Infiltration. PeerJ.

[38] Trink J., Gao B., Li R., Patel J.H., Bajwa U., Zernecke A., Butt E., Krepinsky J.C. (2026). LASP1 Mediates ADAM17 Upregulation in High Glucose to Promote Fibrosis in Diabetic Kidneys. Diabetologia.

[39] Xie P., You Q., Zhu J., Xie W., Wei P., Zhu S., Du Y., Gao X. (2023). PDLIM1 Inhibits Cell Migration and Invasion in Diabetic Retinopathy via Negatively Regulating Wnt3a. Sci. Rep..

[40] Wang L., Han Y., Wang X. (2021). The Relationship between Plasma Serglycin Levels and the Diagnosis of Diabetic Retinopathy. J. Clin. Lab. Anal..

[41] Hasegawa Y., Ishigaki Y. (2017). Heparin Cofactor II: A Novel Plausible Link of Obesity and Diabetes with Thrombosis. J. Atheroscler. Thromb..

[42] Kurahashi K., Inoue S., Yoshida S., Ikeda Y., Morimoto K., Uemoto R., Ishikawa K., Kondo T., Yuasa T., Endo I. (2017). The Role of Heparin Cofactor II in the Regulation of Insulin Sensitivity and Maintenance of Glucose Homeostasis in Humans and Mice. J. Atheroscler. Thromb..

[43] Hara T., Uemoto R., Sekine A., Mitsui Y., Masuda S., Kurahashi K., Yoshida S., Otoda T., Yuasa T., Kuroda A. (2021). Plasma Heparin Cofactor II Activity Is Inversely Associated with Albuminuria and Its Annual Deterioration in Patients with Diabetes. J. Diabetes Investig..

[44] Park J.-H., Park K.-J. (2025). Genetic Variants Associated with Metabolic Dysfunction-Associated Fatty Liver Diseases in a Korean Population. Eur. J. Med. Res..

[45] Wu G., Wang K., Xue Y., Song G., Wang Y., Sun X., Zhong L., Zhou C., Shen B., Chen J. (2016). Association of rs5764455 and rs6006473 Polymorphisms in PARVB with Liver Damage of Nonalcoholic Fatty Liver Disease in Han Chinese Population. Gene.

[46] Mbiandjeu S., Balduini A., Malara A. (2022). Megakaryocyte Cytoskeletal Proteins in Platelet Biogenesis and Diseases. Thromb. Haemost..

[47] Barale C., Russo I. (2020). Influence of Cardiometabolic Risk Factors on Platelet Function. Int. J. Mol. Sci..

[48] Ding Q., Wang F., Guo X., Liang M. (2021). The Relationship between Mean Platelet Volume and Metabolic Syndrome in Patients with Type 2 Diabetes Mellitus: A Retrospective Study: A Retrospective Study. Medicine.

[49] Sadoune M., Mourad J., Luc C., Ragot H., Mateo P., Polidano E., Cohen-Solal A., Dorrifourt S., Li Z., Samuel J.-L. (2025). Qsox1 Contributes to Vascular Remodelling in Response to Hypertension. J. Vasc. Res..

[50] França K.C., Martinez P.A., Prado M.L., Lo S.M., Borges B.E., Zanata S.M., San Martin A., Nakao L.S. (2020). Quiescin/sulfhydryl Oxidase 1b (QSOX1b) Induces Migration and Proliferation of Vascular Smooth Muscle Cells by Distinct Redox Pathways. Arch. Biochem. Biophys..

[51] Zhu X., Hu R., Brissova M., Stein R.W., Powers A.C., Gu G., Kaverina I. (2015). Microtubules Negatively Regulate Insulin Secretion in Pancreatic β Cells. Dev. Cell.

[52] Wittwer J., Bradley D. (2021). Clusterin and Its Role in Insulin Resistance and the Cardiometabolic Syndrome. Front. Immunol..

[53] López-Otín C., Blasco M.A., Partridge L., Serrano M., Kroemer G. (2023). Hallmarks of Aging: An Expanding Universe. Cell.

[54] Franceschi C., Garagnani P., Parini P., Giuliani C., Santoro A. (2018). Inflammaging: A New Immune-Metabolic Viewpoint for Age-Related Diseases. Nat. Rev. Endocrinol..

[55] Spinelli R., Baboota R.K., Gogg S., Beguinot F., Blüher M., Nerstedt A., Smith U. (2023). Increased Cell Senescence in Human Metabolic Disorders. J. Clin. Investig..

[56] Shimizu I., Yoshida Y., Suda M., Minamino T. (2014). DNA Damage Response and Metabolic Disease. Cell Metab..

[57] López-Polo V., Maus M., Zacharioudakis E., Lafarga M., Attolini C.S.-O., Marques F.D.M., Kovatcheva M., Gavathiotis E., Serrano M. (2024). Release of Mitochondrial dsRNA into the Cytosol Is a Key Driver of the Inflammatory Phenotype of Senescent Cells. Nat. Commun..

[58] Gaya da Costa M., Poppelaars F., van Kooten C., Mollnes T.E., Tedesco F., Würzner R., Trouw L.A., Truedsson L., Daha M.R., Roos A. (2018). Age and Sex-Associated Changes of Complement Activity and Complement Levels in a Healthy Caucasian Population. Front. Immunol..

[59] Qian Y., Yang L., Chen J., Zhou C., Zong N., Geng Y., Xia S., Yang H., Bao X., Chen Y. (2024). SRGN Amplifies Microglia-Mediated Neuroinflammation and Exacerbates Ischemic Brain Injury. J. Neuroinflamm..

[60] Doncheva A.I., Norheim F.A., Hjorth M., Grujic M., Paivandy A., Dankel S.N., Hertel J.K., Valderhaug T.G., Böttcher Y., Fernø J. (2022). Serglycin Is Involved in Adipose Tissue Inflammation in Obesity. J. Immunol..

[61] Lacolley P., Regnault V., Avolio A.P. (2018). Smooth Muscle Cell and Arterial Aging: Basic and Clinical Aspects. Cardiovasc. Res..

[62] Aihara K.-I., Azuma H., Takamori N., Kanagawa Y., Akaike M., Fujimura M., Yoshida T., Hashizume S., Kato M., Yamaguchi H. (2004). Heparin Cofactor II Is a Novel Protective Factor against Carotid Atherosclerosis in Elderly Individuals. Circulation.

[63] Statzer C., Park J.Y.C., Ewald C.Y. (2023). Extracellular Matrix Dynamics as an Emerging yet Understudied Hallmark of Aging and Longevity. Aging Dis..

[64] Rifkin D., Sachan N., Singh K., Sauber E., Tellides G., Ramirez F. (2022). The Role of LTBPs in TGF Beta Signaling. Dev. Dyn..

[65] Ren L.-L., Miao H., Wang Y.-N., Liu F., Li P., Zhao Y.-Y. (2023). TGF-β as A Master Regulator of Aging-Associated Tissue Fibrosis. Aging Dis..

[66] Ilani T., Alon A., Grossman I., Horowitz B., Kartvelishvily E., Cohen S.R., Fass D. (2013). A Secreted Disulfide Catalyst Controls Extracellular Matrix Composition and Function. Science.

[67] Ilani T., Reznik N., Yeshaya N., Feldman T., Vilela P., Lansky Z., Javitt G., Shemesh M., Brenner O., Elkis Y. (2023). The Disulfide Catalyst QSOX1 Maintains the Colon Mucosal Barrier by Regulating Golgi Glycosyltransferases. EMBO J..

[68] Liu Y., Buil A., Collins B.C., Gillet L.C.J., Blum L.C., Cheng L.-Y., Vitek O., Mouritsen J., Lachance G., Spector T.D. (2015). Quantitative Variability of 342 Plasma Proteins in a Human Twin Population. Mol. Syst. Biol..

[69] Geyer P.E., Hornburg D., Pernemalm M., Hauck S.M., Palaniappan K.K., Albrecht V., Dagley L.F., Moritz R.L., Yu X., Edfors F. (2024). The Circulating Proteome─Technological Developments, Current Challenges, and Future Trends. J. Proteome Res..

[70] Peng J., Zhang H., Niu H., Wu R. (2020). Peptidomic Analyses: The Progress in Enrichment and Identification of Endogenous Peptides. Trends Anal. Chem..

[71] Nazziwa J., Freyhult E., Hong M.-G., Johansson E., Årman F., Hare J., Gounder K., Rezeli M., Mohanty T., Kjellström S. (2024). Dynamics of the Blood Plasma Proteome during Hyperacute HIV-1 Infection. Nat. Commun..

[72] Anderson N.L., Anderson N.G. (2002). The Human Plasma Proteome: History, Character, and Diagnostic Prospects: History, Character, and Diagnostic Prospects. Mol. Cell. Proteom..

[73] Carrasco-Zanini J., Wheeler E., Uluvar B., Kerrison N., Koprulu M., Wareham N.J., Pietzner M., Langenberg C. (2024). Mapping Biological Influences on the Human Plasma Proteome beyond the Genome. Nat. Metab..

[74] Tammen H., Schulte I., Hess R., Menzel C., Kellmann M., Mohring T., Schulz-Knappe P. (2005). Peptidomic Analysis of Human Blood Specimens: Comparison between Plasma Specimens and Serum by Differential Peptide Display. Proteomics.

[75] Yi J., Kim C., Gelfand C.A. (2007). Inhibition of Intrinsic Proteolytic Activities Moderates Preanalytical Variability and Instability of Human Plasma. J. Proteome Res..

[76] Farinas M.F., Chen Y., Zeng X., Nafash M.N., Cohen A.D., Lopez O.I., Karikari T.K. (2025). Plasma vs. Serum: Which Is Better for Proteomic Blood Biomarker Analysis? Evaluation of the Novel NULISA Platform. medRxiv.

[77] Shraim R., Diorio C., Canna S.W., Macdonald-Dunlop E., Bassiri H., Martinez Z., Mälarstig A., Abbaspour A., Teachey D.T., Lindell R.B. (2025). A Method for Comparing Proteins Measured in Serum and Plasma by Olink Proximity Extension Assay. Mol. Cell. Proteom..

[78] Shi T., Su D., Liu T., Tang K., Camp D.G., Qian W.-J., Smith R.D. (2012). Advancing the Sensitivity of Selected Reaction Monitoring-Based Targeted Quantitative Proteomics. Proteomics.

[79] Carr S.A., Abbatiello S.E., Ackermann B.L., Borchers C., Domon B., Deutsch E.W., Grant R.P., Hoofnagle A.N., Hüttenhain R., Koomen J.M. (2014). Targeted Peptide Measurements in Biology and Medicine: Best Practices for Mass Spectrometry-Based Assay Development Using a Fit-for-Purpose Approach. Mol. Cell. Proteom..

